# The physiological function of long-noncoding RNAs

**DOI:** 10.1016/j.ncrna.2020.09.003

**Published:** 2020-09-17

**Authors:** He Chen, Ge Shan

**Affiliations:** CAS Key Laboratory of Innate Immunity and Chronic Disease, CAS Center for Excellence in Molecular Cell Science, School of Life Sciences, University of Science and Technology of China, Hefei, Anhui Province, 230027, China

**Keywords:** Long noncoding RNA, LincRNA, CircRNA, CRISPR, Phenotype, Physiological function

## Abstract

The physiological processes of cells and organisms are regulated by various biological macromolecules, including long-noncoding RNAs (lncRNAs), which cannot be translated into protein and are different from small-noncoding RNAs on their length. In animals, lncRNAs are involved in development, metabolism, reproduction, aging and other life events by *cis* or *trans* effects. For many functional lncRNAs, there is growing evidence that they play different roles on cellular level and organismal level. On the other hand, many annotated lncRNAs are not essential and could be transcription noises. In this minireview, we investigate the physiological function of lncRNAs in cells and focus on their functions and functional mechanisms on the organismal level. The studies on lncRNAs using different classic animal models such as worms and flies are summarized and discussed in this article.

## 1 Introduction

**Fig. 1:**
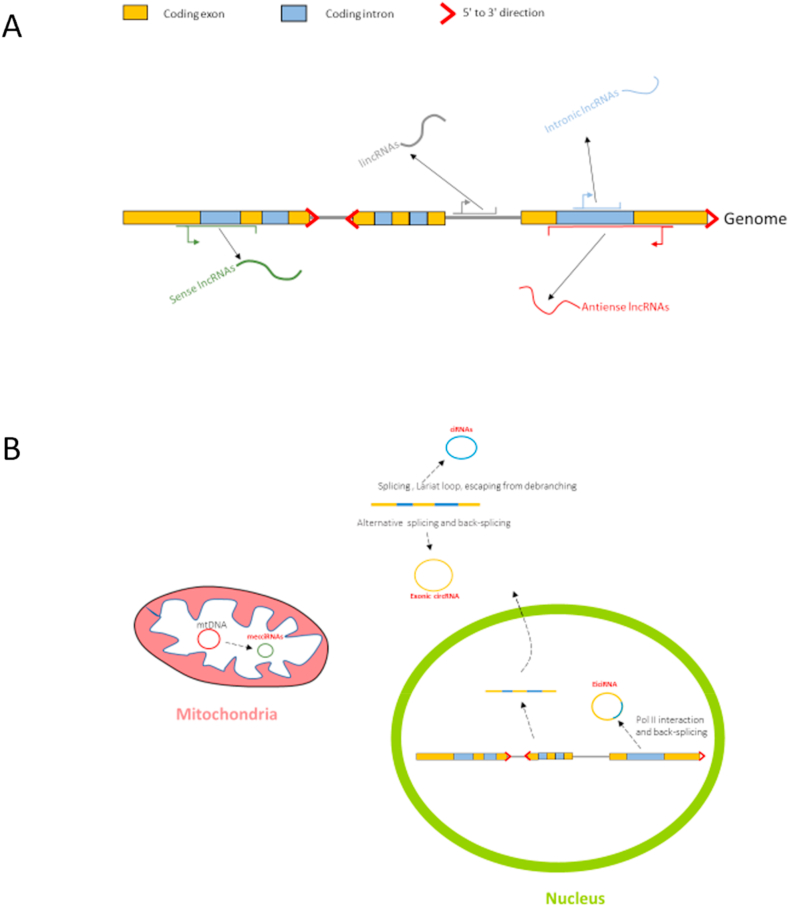
Types of long-noncoding RNA(linear and circular). (A) 4 groups of lncRNA according to the distribution of lncRNAs in the genome, relative positions with nearby coding genes and transcription directions. (B) 4 groups of circRNA in cytoplasm, nucleus and mitochondria.

Recent studies illustrate varieties of noncoding RNAs which lack ORFs and cannot translate. Noncoding RNAs can be classified according to their location, function, size or secondary structure [1,2]. The noncoding RNAs with the length of hundreds (200) nt or more are long(large) noncoding RNAs (LncRNAs) [3,4]. LncRNAs can be linear or circular, distributed in the nucleus, cytoplasm and mitochondria [[5], [6], [7], [8]](Fig. 1). According to the distribution of lncRNAs in the genome, relative positions with nearby coding genes and transcription directions, they can be divided into four groups: long intervening/intergenic noncoding RNAs(lincRNAs), intronic lncRNAs, sense lncRNAs and antisense lncRNAs [3]. According to their functions, lncRNAs can be roughly divided into: functional lncRNAs, whose transcripts can regulate genes expression *in cis* or *in trans*; lncRNAs play their roles during transcription, but their transcripts have no functions; no functions lncRNAs which might be transcription noises [[9], [10], [11]].

In cells, lncRNAs which located in the nucleus or cytoplasm interact with DNAs, proteins or other RNAs. They involve in the cell's proliferation, differentiation and apoptosis [12]. In animals, lncRNAs play their functional roles in development, reproduction, aging and disease [[12], [13], [14]]. Interestingly, in some cases, the key roles of lncRNAs in cells do not match their importance at the whole-organism scale, for example, knockout almost all lincRNAs one by one in nematodes did not exhibit critical phenotypes [15]. In mammals, a widely studied long noncoding RNA, *Hotair* regulates epidermal cell differentiation and interacts with epigenetic factors such as PRC2 to participate in tumor metastasis [16,17]. Although *Hotair* plays a key role in cultural cells, one study suggested that *Hotair* were dispensable for whole animals [18]. *Hotair* knockout mice did not exhibit the expecting phenotypes suggesting they are not necessary for mouse development and embryonic survival [18]^.^ The same also occurred in the study of *Malat1* [[19], [20], [21], [22]].With the advances of technology, especially CPRISPR gene editing and Next-generation sequencing, a growing body of lncRNA researches are conducted at the whole-organism scale. In this review, we summarize the physiological functions and mechanisms of lncRNAs in cells and animal models.

## 2 Cellular physiological functions of LncRNAs

### 2.1 lincRNAs

Long intervening/intergenic noncoding RNAs(lincRNAs) are lncRNAs located between coding genes and have no overlap with any annotated protein-coding sequences. LincRNAs exercise physiological functions in cells such as carcinogenesis, infection and inflammation. For example, *NEAT* (Nuclear Enriched Abundant Transcript) RNAs including *Neat1* and *Malat1*(also named as *Neat2*), are classic examples of lincRNAs in mammalian cells and are involved in carcinogenesis [[23], [24], [25], [26], [27]]. They are conserved across various mammals and locate in the nuclear and participate in many biological processes such as paraspeckle formation, cell cycle regulation, alternative splicing and cancer cells migration [28,29]. Another extensively studied lincRNA *Hotair* which located in *HOXC* gene cluster, is a highly expressed gene in metastatic breast cancers [30,31]*. Hotair* interacts with Polycomb-group proteins and reprogram chromatin state *in trans* [32,33]. *LincRNA-p21* activated by tumor suppressor P53, is another important transcriptional repressor that binds to the hnRNP family protein hnRNP-K after DNA damage and participates in maintaining of the P53 induced genome stability [34]. Not only lincRNAs could be taken as regulators and biomarkers in tumor cells, but also functional molecules for cellular physiology. For instance, lincRNAs with elevated expression patterns in iPS and ES cells suggest their functions in establishment and maintenance of pluripotency [35,36]. In pluripotent stem cells, *lincRNA-RoR* modulates reprogramming as the direct targets of key transcription factors [35]. In other studies, lincRNAs played their roles in immunomodulation such as *lincRNA-EPS.* It acts as an important regulator of immune response genes in immune cells *in trans* [37].

### 2.2 Circular RNAs

Not only linear but also circular long noncoding RNAs modulate cellular physiological processes [[38], [39], [40]]. Circular RNAs are generated by back-splicing from precursor mRNAs and displayed special expression pattern in tissues and developmental stages [[41], [42], [43], [44]]. In general, circular RNAs are roughly divided into four categories: Exonic circRNA, circular intronic RNAs (ciRNAs), exon–intron circRNAs (EiciRNAs) and mitochondria-encoded circRNA(mecciRNAs) [7,8,45,46]. Circular RNAs can function as microRNA sponges and regulate genes expression *in trans* [47,48]. For example, *CDR1as*(*ciRS-7*) can bind *miR-7* and *miR-671* to regulate the expression of their target genes and functions in cellular proliferation and apoptosis [[49], [50], [51]]. *CDR1as* knockout mice displayed abnormal brain function due to the defect of synaptic neurotransmission [52]. *CDR1as* is also a regulator of insulin secretion and oncogene [53,54]. On the other hand, Circular RNAs can act as protein sponge, decoy or scaffold [55,56]. For example, *Cia-cGAS* acts as nuclear cGAS sponge to block its enzymatic activity in hematopoietic stem cells to protect their homeostasis [55]. In another research, *circ-Foxo3* constructs *circ-Foxo3*-p21-CDK2 complex to block cell cycle progression by suppressing CDK2 [56]. Circular RNAs are also able to function *in cis*. Take EiciRNAs as an example, which are circularized with introns “retained” between their exons [7]. EiciRNAs such as *circEIF3J* and *circPAIP2* can hold U1 snRNP by specific RNA-RNA interaction, then the complexes further interact with the Pol II at the promoters of parental genes to enhance their expression level and arise a positive feedback in genes expression [7]. In addition to the nuclear genome, the circular RNAs encoded by mitochondrial genome which are termed as mecciRNAs also have important roles [8].The mecciRNAs promote mitochondrial importation of nuclear-encoded proteins, by interacting with TOM40 and PNPASE serving as molecular chaperones [8]. Dynamic expression of *mecciND1* under stress regulated cellular physiology by increasing the RPA70 and RPA32 protein levels in mitochondria [8].

### 2.3 Other LncRNAs

In addition to lincRNAs, there are other forms of lncRNAs which modulate cellular physiological functions, such as antisense lncRNAs and long intronic noncoding RNAs [[57], [58], [59], [60], [61]]. For example, nuclear-enriched *AS Uchl* RNA in dopaminergic neurons upregulates UCHL1 protein levels via the SINEB2 repeat element [57]. The inhibition of mTORC1 by rapamycin increased UCHL1 protein levels by *AS Uchl* implied a mechanism of antisense lncRNAs in the control of cellular stress signaling pathways and their roles in neurodegenerative diseases [57]. In other researches, long intronic transcripts take their roles as precursors of small RNAs, cofactors of alternative promoters and regulators of alternative pre-mRNA splicing [58]. Take *SAF* as an example, a 1500 nt intronic lncRNA transcribed from the opposite strand of FAS gene intron 1 regulates the alternative splicing of FAS *in cis* to protect cells from membrane-mediated apoptosis [59]. In addition, overlapped transcripts are also involved in cellular events such as *5S-OT*(5S rRNA overlapped transcript) [60]. *5S-OT* modulates 5S rRNA transcription in mice and humans by *cis* effect, and it is intriguing that human *5S-OT* regulates alternative splicing of numerous genes by U2AF65 and Alu pairing *in trans* [60]. In a classic human macrophage differentiation model, knockdown of *5S-OT* decreased THP-1 cells differentiation efficiency [60]. LncRNAs also participate in cell division, for example, 171 nt *α-satellite* RNAs which are transcribed from centromeric repeats are managed by a RNAi pathway and function in chromosome segregation [61].

## 3 The physiological functions of LncRNA in animals

### 3.1 Caenorhabditis elegans

As simplest one of animal models, *Caenorhabditis elegans* has many advantages in genetics and molecular biology researches [[62], [63], [64], [65]]. Using *C. elegans*, researchers found *rncs-1*, an 800 nt lincRNA(long intervening noncoding RNAs) is up-regulated after starvation which is expressed in intestine and hypodermis, and inhibits Dicer cleavage *in vitro* and *in vivo* [66].Overexpression of *rncs-1* led to an increased frequency of males during starvation indicated its functional roles of lncRNAs in response to stress [66]. LncRNAs can regulate development and sexual maturation. *Lep-5*, a 600 nt cytoplasmic lincRNA, regulates developmental timing as a scaffold to bring LEP-2 into its target gene LIN-28, and takes part in tail tip morphogenesis of males regulating sexual maturation cell-autonomously in nervous system [67,68]. *Lep-5* is conserved across *Caenorhabdit*is uncovering evidence for its function in evolution [67,68].

As model organism which can be handled easily, *C. elegans* is used in resource research [69]. Using available RNA-seq and other techniques, 170 lincRNAs and 60 ancRNAs(antisense lncRNAs) were identified in *C. elegans* [70]. LincRNAs of *C. elegans* are expressed in a stage-specific manner, and many of them are dauer stage-specific or sperm-specific molecules [15,70]. To investigate their spatiotemporal expression, transgenic reporter strains and RNA-seq were generated showing that the expression patterns of lincRNAs are more specific and heterogeneous than transcription factors [71]. LincRNAs of *C. elegans* can be detected in different developmental stages and tissues including intestine cells, hypodermal cells, muscles and neurons [71]. Using CPRISPR knockout strains, the functions of lincRNAs in *C. elegans* were systematically evaluated by our group, several representative phenotypes were tested in these lincRNAs KO animals, and the global features such as their exon numbers, conservation, and length were described [15]. 23 of 155 KO mutants showed minor abnormalities in locomotion, defecation, pharyngeal pumping, egg retention, development and offspring numbers. Mechanistically, some of these lincRNAs played *cis* roles to regulate the expression neighboring genes, some of them could function as ceRNAs against microRNAs *in trans*. By bioinformatics analysis from ChIP-seq datasets (modENCODE), the 23 phenotypic lincRNAs are regulated by more transcription factors than the others indicating that lincRNAs are targets of TFs in neurons to control their function directly [15,72,73].

### 3.2 Drosophila melanogaster

Like *C. elegans*, *Drosophila melanogaster* is also a kind of classic and simple animal model but have more observable phenotypes in genetics [[74], [75], [76], [77]]. *Drosophila* is taken as a research platform to investigate the *in vivo* functions of noncoding RNAs for decades [[78], [79], [80], [81]]. Transcription of many *Drosophila* lncRNAs occurs during embryogenesis and display spatiotemporally expression [78]. As a resource study, *Wen* et al. identified 128 testis-specific lncRNAs in which 105 of them were knocked out by CRISPR. Among the KO mutants, only 33 (31%) exhibited male-specific fertility defects most of them (32) just have partially decreased male fertility [82].

One of the important functions of lncRNAs is to regulate the chromatin state. LncRNAs involving in the X chromosome dosage compensation were elucidated in the studies of *Drosophila* [83]. *RoX1* and *roX2* genes produce male-specific lncRNAs that co-localize with the MSL (Male-Specific Lethal) protein complex. They form a stable association with the protein complex and activate the expression of X-linked genes in males to equalize genes expression between two sexes [83]. The ChIRP (Chromatin Isolation by RNA Purification) -seq analysis displayed the *Drosophila roX* genes binding sites on X chromosome directly [84]. Interestingly, both *roX1* and *roX2* are non-essential. Deletion *roX1* or *roX2* in both sexes had no significant phenotypes [83]. Males of *roX* double mutants were disrupted in development. Males carrying *roX* - chromosomes were lethal and only 5% of them were survival. Although the double mutant showed the male-specific lethal phenotypes, the females of them were not affected, either *roX1* or *roX2* cDNAs could rescue the male-specific phenotypes of the double mutants [83]. At molecular level, *roX1* and *roX2* intact with some important proteins (MSL1-3, MOF, MLE) and form MSL complex to regulate epigenetic modification such as histone acetylation [85].

Evidence in *Drosophila* shows that lncRNAs participate in the cellar response to stress. For example, one of the heat shock proteins *hsr-omega* encoding a nuclear lncRNA, participates in the reorganization of nucleoplasmic omega speckles after heat shock [86]. It functions as a hub and accumulate hnRNPs. *Hsr-omega* nullisomic mutants resulted in embryonic lethality [[86], [87], [88]]. Recent studies also demonstrated the regulation of lncRNAs in neurogenesis and their molecular mechanism in flies [89,90]. Neurogenic lncRNAs are expressed specifically during early stages of nervous system development and mark specific subsets of neurogenic cell types including neurons and glia [89]. Another study indicated that lncRNAs controlled by Hox genes participated in the formation of anteroposterior (AP) axis of *Drosophila*. A 92k nt lncRNA encoded by the intergenic region isolating Abd-A and Abd-B was identified [90]. This CNS-specific lincRNA(*iab8ncRNA*) suppresses the expression of Abd-A genes by two redundant mechanisms: the first way is mediated by *mir-iab-8*, a microRNA encoded by the intronic sequence within *iab8ncRNA*; on the other hand, the transcriptional interference by *iab8ncRNA* on Abd-A promoter is involved in the regulation [90].In addition, lncRNAs act not only in the formation and function of nervous system but also in the behavior of *Drosophila* [81,91,92].For instance, the cytoplasmic *yellow-achaete* intergenic RNA (*yar*) which is conserved in *Drosophila* regulates the sleep behavior, the phenotypic rescue by a *yar* transgene suggests that it functions *in trans* [81].

### 3.3 Zebrafish

Compared to simple models such as nematodes and flies, *Danio rerio*(zebrafish) belongs to vertebrates and is closer to mammals. Zebrafish is one of the most classic model vertebrates [[93], [94], [95]]. It has many features such as ease of feeding and embryo transparency that make it an excellent model for research of developmental biology, stem cell research, physiology and toxicology [[96], [97], [98], [99]].LncRNAs of *zebrafish* were identified using RNA deep sequencing approaches in three independent studies resent years [100]. *Ulitsky* et al. annotated 567 lincRNAs, by using RNA-seq, ploy(A) mapping and chromatin marks. Among them, only 29 had putative mammalian orthologs, but most of them displayed tissue-specific expression [101]. Using MO (morpholino antisense oligos) knockdown protocol, two conserved lincRNAs exhibited functional roles and the MO resulted in embryonic defects: *Linc-oip5* was required for the normal size of head, eyes and tail; *linc-birc6* was required for brain and eyes development [101]. *Pauli* et al. performed RNA-seq experiments at 8 developmental time points of zebrafish and identified lncRNAs expressed during embryogenesis [102]. LncRNAs of zebrafish were expressed at lower levels but in narrower time windows compared with coding genes in early embryos and showed tissue-specific and subcellularly restricted expression patterns [102]. By RNA-seq of 5 different tissues from adult zebrafish, *Kaushik* et al. annotated 442 predicted lncRNAs with 419 were newly annotated [103]. 77 lncRNAs were tissue-specific and the adult brain enriched the most tissue-specific lncRNAs [103].

Some evidence suggests that not only lincRNAs regulate the development of zebrafish, but also antisense lncRNAs [102,104,105]. In zebrafish, an antisense lncRNA, *tie-1AS* which is expressed spatiotemporally can bind *tie-*1 mRNA selectively to form *tie-1: tie-1AS* hybrid to regulate *tie-1* transcript levels [104]. Overexpression of *tie-1AS* led to defects in the formation of contact junctions in endothelial cells and abnormal vascular development. In addition, *tie-1AS* is conserved in humans and mice [104,106]. As vertebrates, zebrafish is used for a model to reveal the conserved functional lncRNAs in humans and their roles in diseases [[106], [107], [108]]. The roles of lncRNAs in the regulation of sexual reproduction and behavior were analyzed by *Yuan* et al. [109]. In the brain of zebrafish, there were numerous gender-specific lncRNAs like humans with 12 new lncRNAs were annotated [109]. Even though several lncRNAs may be critical and essential in fish, a more recently resource study using CRISPR KO mutants indicated that the majority of individual lncRNAs in zebrafish had no key roles, and the phenotypes of the KO mutants such as embryogenesis, viability and fertility had no overt abnormalities [110].

### 3.4 Mammals

The atlas of biological functions of several “star” lncRNAs is drawing both *in vivo* and *in vitro*, using both cultured cells and mammalian models such as mice and rats [[111], [112], [113], [114]]. *Mus musculus*(mouse) were usually utilized as a mammalian model in genetics and molecular biology for decades [115]. Mice and humans share more than 90% conserved regions in the genomes, but in the transcription level, lncRNAs are expressed at a lower level and less conservation in sequences comparing with coding genes. However, there are thousands of conserved orthologous lncRNAs [116]. The X-chromosome dosage in mammals is controlled by a long noncoding RNA, *Xist* [117,118]. It is similar to *roX* genes in *Drosophila*, but *Xist* effects in an opposite way: *roX1* and *roX2* activate X-linked genes in males, however *Xist* inactivate X-linked genes in females in embryonic development [[119], [120], [121]]. *Xist* RNA can coat and accumulate on one X chromosome (where it is expressed), recruit a series of epigenetic regulators then transcriptional silencing rapidly ensues [122,123]. Mutations of *Xist* in mice result in females embryonic lethal inheriting paternal allele but males without any phenotypes [124,125]. Another lncRNA which was discovered for several decades is *H19*, a 2.5 kb untranslated transcript from the distal region of chromosome 7 in mice [126]. It is expressed at a very high level in embryonic tissues including endoderm and mesoderm, and its expression level maintains during several days after birth then disappears in adult [117,118]. *H19* is an imprinted and exclusive maternal origin allele gene [[127], [128], [129]]. The deletion of *H19* in mice led to no obvious phenotypes except slightly increased growth in homozygous mutants [124,129]. For other widely studied lncRNAs, for example, *Neat1* and *Malat1* are globally expressed and have cellular functions, the mutant mice do not exhibit overt abnormalities except for the defects of paraspeckles [130,131]. In another study, the KO mice of *Hotair* were fertile and viable with slight skeletal abnormalities [132].

For the whole organism, there are several lncRNAs which are essential in mammals. In one study, the function of 18 mammalian lincRNAs candidates were evaluated by mice mutants [22]. 3(*Fendrr*, *Peril*, and *Mdgt*)of these were critical and the mutants displayed embryonic and postnatal lethal phenotypes. *Fendrr* and *mdgt* might have functions in multiple organs, and *Peril* might have functions in ESCs (embryonic stem cells) [22]. However, most of lncRNAs are not essential for their loss-of-function mutants are viable and fertile [22,124]. But on the other hand, they in turn participate in the regulation of many physiological and pathological processes [133,134]. LncRNAs are involved in pathogen infection [135,136]. For example, *Peng* et al. reported that the mice infected by SARS-CoV showed significant different expression of lncRNAs which were similar to influenza virus infection [135]. LncRNAs are also involved in the cellular responses to bacterial infection such as *Sros1* which could sensitize mice to *L. monocytogenes* [136]. LncRNA also play their roles in cancers [137]. *Malat-1* is named for its function in metastasis of lung cancer cells, deletion of *malat-1* in mice impaired tumor cells metastasis. *Malat-1* could also be taken as a predictive marker clinically [137]. LncRNAs are involved in metabolism [138]. For instance, lipid metabolism regulated by lncRNAs associates with obesity and hepatic steatosis, *Muret* et al. summarized 60 lncRNAs in mice and humans involved in lipid metabolism and their functions in diseases [139].Additionally, there is evidence that lncRNAs can be regulator in neuroregeneration suggesting their roles in neurodegenerative diseases [140]. *Perry* et al. reported the lncRNAs expressed during neuroregeneration in dorsal root ganglia of mice and found two key lncRNAs, *Silc1* and *Norris1* [141]. *Silc1* regulated transcription factor Sox11 *in cis*, *Silc1* KO mice displayed delayed regeneration following injury [141].

## 4 Summary

**Fig. 2:**
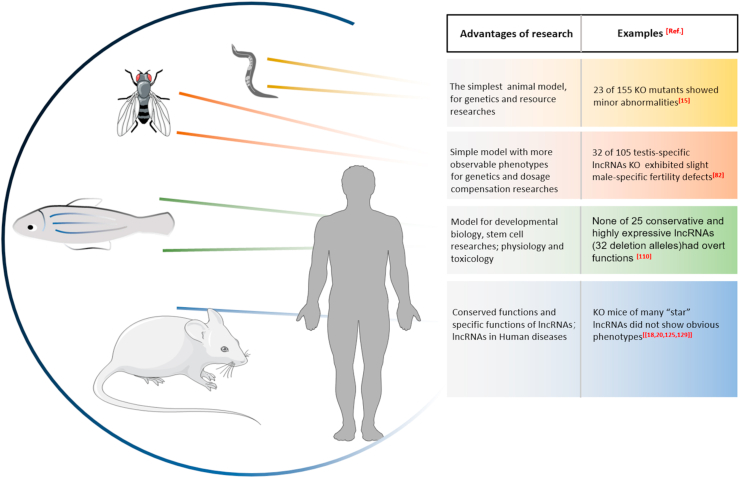
LncRNAs Researches in animal models. Advantages and typical cases of lncRNAs researches by different models are listed. In most cases, lncRNAs are not essential for whole animals.

For cellular physiology, lncRNAs function in proliferation, differentiation, stress, aging, and apoptosis by epigenetic, transcriptional, and post-transcriptional regulation. They can be various forms and play *trans* and *cis* roles in cells [5,10]. The functions and functional mechanism of lncRNAs in physiology of animals are revealed using classic animal models [62,74,93]. Much evidence exhibits the differences of their effects between cellular levels and whole organism levels. Even though lncRNAs show their important roles in many biological processes, depletion of them impact fewer phenotypes than expected [124]. The resource research using C. elegans, Drosophila and zebrafish suggest that lncRNAs are not essential for whole animal in most cases [15,82,110]. Interestingly, the KO mice of many “star” lncRNAs do not show obvious phenotypes [18,20,125,129] (Fig. 2). However, lncRNAs may play their functional roles under particular physiological and pathological conditions, making them potential key molecule in organisms.

## Declaration of competing interest

All the authors declared that they have no conflicts of interest to this work. We declare that there is no professional or other personal interest of any nature or kind in any product, service and/or company that could be construed as influencing the position presented in, or the review of, the manuscript entitled.
